# Copy number analyses of DNA repair genes reveal the role of poly(ADP-ribose) polymerase (PARP) in tree longevity

**DOI:** 10.1016/j.isci.2021.102779

**Published:** 2021-06-24

**Authors:** Yuta Aoyagi Blue, Junko Kusumi, Akiko Satake

**Affiliations:** 1 Graduate School of Systems Life Sciences, Kyushu University, 744 Motooka, Fukuoka, 819-0395, Japan; 2 Department of Environmental Changes, Faculty of Social and Cultural Studies, Kyushu University, 744 Motooka, Fukuoka819-0395, Japan; 3 Department of Biology, Kyushu University, 744 Motooka, Fukuoka819-0395, Japan

**Keywords:** Biological sciences, Phylogenetics, Plant genetics, Plant biology

## Abstract

Long-lived organisms are exposed to the risk of accumulating mutations due to DNA damage. Previous studies in animals have revealed the positive relationship between the copy number of DNA repair genes and longevity. However, the role of DNA repair in the lifespan of plants remains poorly understood. Using the recent accumulation of the complete genome sequences of diverse plant species, we performed systematic comparative analyses of the copy number variations of DNA repair genes in 61 plant species with different lifespans. Among 121 DNA repair gene families, *PARP* gene family was identified as a unique gene that exhibits significant expansion in trees compared to annual and perennial herbs. Among three paralogs of plant *PARPs*, *PARP1* showed a close association with growth rate. PARPs catalyze poly(ADP-ribosyl)ation and play pivotal roles in DNA repair and antipathogen defense. Our study suggests the conserved role of PARPs in longevity between plants and animals.

## Highlights

• Comparing the copy number variations of DNA repair genes in diverse plant species
• *PARP* gene family showed higher copy number in trees compared to herbs
• There was negative correlation between copy number of *PARP1* and growth rate in trees
• Increased copy number of *PARP* would be evolutionary favored in plant longevity

## Graphical abstract

**Figure:**
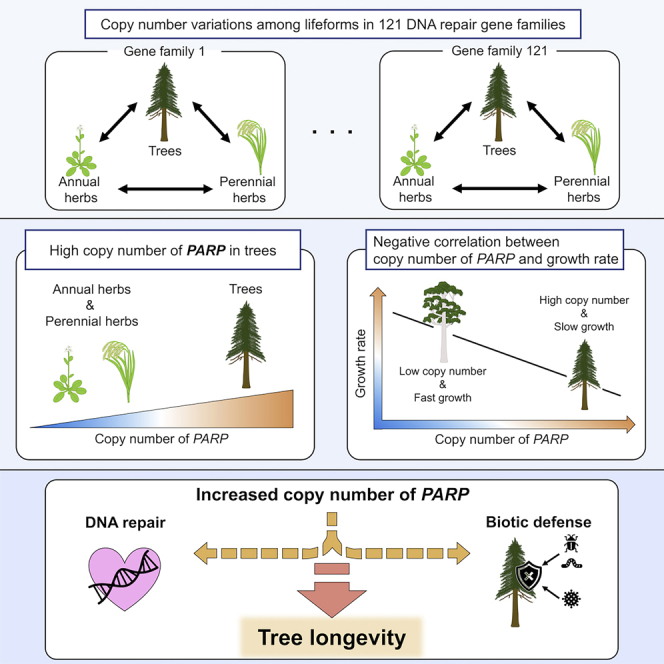

## Introduction

Organisms accumulate DNA damage via exogenous environmental factors (e.g., ionizing radiation and UV light) and constant threats to the endogenous metabolic process (e.g., production of reactive oxygen species and errors in DNA metabolism). DNA lesions commonly include oxidized or alkylated base damage, single- and double-strand breaks, intra- or inter-strand crosslinks, and base loss. The resulting alteration of the DNA structure leads to genomic instability, apoptosis, or senescence, which can affect the organism’s development and aging process. To reverse the potentially deleterious damage, life in all its forms has evolved sophisticated machinery, involving hundreds of proteins, to efficiently recognize and properly repair DNA damage.

Depending on the type of DNA lesion, organisms have developed diverse functional pathways for DNA repair (Sancar et al., 2004; Ciccia and Elledge, 2010). The base excision repair (BER) and direct damage reversal/repair (DR) pathways repair DNA base damage, whereas mismatch repair (MMR) corrects base mispairs and small loops often found in repetitive sequence DNA. More complex lesions, such as pyrimidine dimers and intrastrand crosslinks, are corrected by nucleotide excision repair (NER). Double-strand breaks (DSBs) are repaired either by non-homologous end-joining (NHEJ) or homologous recombination (HR). These major functional pathways for DNA repair have been identified in virtually all organisms, including bacteria, archaea, and eukaryotes, reflecting the universal need to counter DNA damage in living organisms (Aravind et al., 1999; Eisen and Hanawalt, 1999).

With the recent accumulation of the complete genome sequences of diverse organisms, it has become possible to systematically compare the DNA repair systems of the respective organisms and identify the origins of the different repair genes and functional pathways. A global comparative analysis of DNA repair proteins based upon the available complete genome sequences of bacteria, archaea, and eukaryotes has shown that repair machinery shows considerable diversity in terms of the presence and absence of genes. Eisen and Hanawalt (1999) showed that only DR pathways are highly homologous between species (they make use of homologous genes in all species), whereas other pathways are not homologous, with the use of genes of different origins between species despite performing the same functions.

The diversity of repair machinery among species can be formed by frequent gene duplication and gene loss. Members of the *recA*/*RAD51* gene family, which is associated with HR, are suggested to be generated by multiple duplication events (one before the archaea/eukaryote split and another in the early stage of eukaryotic evolution), gene loss, and endosymbiotic gene transfer (Lin et al., 2006). A study based on angiosperm genomes reported the strong selection pressure to preserve many of the DNA repair genes as singletons in *Arabidopsis thaliana*, regardless of repeated whole genome or single gene duplication events in flowering plants (De Smet et al., 2013). The species-specific history of gene duplication and loss will result in copy number variations of DNA repair genes among species, which can have profound effect on organismal phenotypes, including mutation rates (Baer et al., 2007), lifespan (Lorenzini et al., 2009; Freitas and De Magalhães, 2011), and adaptation to extreme environments (Matic et al., 1995; White et al., 1999).

Previous studies focused on aging have highlighted the positive correlation of an increased copy number of DNA repair genes and longevity in mammals (Tian et al., 2017). The naked mole-rat, the longest-lived rodent, has higher copy numbers of genes for CCAAT/enhancer binding protein-γ (*CEBPG*), a regulator of DNA repair, and TERF1-interacting nuclear factor 2 (*TINF2*), a protector of telomere integrity compared to more short-lived species (MacRae et al., 2015a). Another long-living mammal, the African elephant, encodes 20 copies of the tumor suppressor gene, *TP53*, which induce apoptosis or senescence programs in response to DNA damage (Sulak et al., 2016). Analyses of genomes of other long-lived species, the bowhead whale and bat, showed the signature of positive selection of multiple DNA repair and DNA damage signaling genes (Zhang et al., 2013a; Keane et al., 2015). These studies in mammals suggest the importance of genome maintenance mechanisms for longevity.

Despite the wealth of studies in animals, there are no studies that employ comparative genome analyses to identify the DNA repair genes associated with the evolution of longevity in plants (Umeda et al., 2021). Plants exhibit a wide range of lifespans, from a few weeks in monocarpic annuals to as long as millennia in long-lived perennials. Plant development fundamentally differs from that of animals. Plant lifespan is characterized by rudimentary body plan, modular growth, and disparity between cell death and death of the organism (Watson and Riha, 2010), allowing high plasticity and indeterminate growth of vegetative meristems that are unique to plants. In perennials, meristematic cells may undergo thousands of divisions. In addition, being sessile organisms, environmental stress may result in increased DNA damage. It is a major interest, therefore, to determine the efficiency of the DNA repair mechanisms in long-lived plant species.

Thanks to the significant progress in the elucidation of the DNA damage repair systems in *A. thaliana* as a model (Hays, 2002; Manova and Gruszka, 2015; Bray and West, 2005; Yoshiyama et al., 2013), all major DNA repair pathways have been reported to be conserved between plants and other organisms. Moreover, a growing number of sequenced genomes in non-model plant species are available. In this study, using more than 60 species of plants, including long-lived trees, perennial herbs, annual herbs, and algae, we performed systematic comparative analyses of the copy number variations of genes that encode proteins involved in DNA repair in diverse plant species with different life forms.

## Results

### Interspecies comparison of copy number ratio of 121 DNA repair gene families

To compare the copy number variations of DNA repair genes between diverse species, we used the PLAZA database (Dicots PLAZA 4.0; Van Bel et al., 2018 and Gymno PLAZA 1.0; Proost et al., 2009), the genomic database of diverse plant species. We used 61 plant species, including 23 tree species, 15 perennial herb species, 21 annual herb species, and two algae species for our analyses (Table 1), thereby covering both angiosperms and gymnosperms. Because the species with large genome sizes would have a large number of DNA repair genes, the PLAZA database provided the normalized index, namely the copy number ratio, by dividing the actual copy number of genes within each gene family in the focal species by the total number of genes in the species (see STAR Methods section). We selected 171 genes involved in DNA repair within *A. thaliana* (Table S1). We used the orthologous groups predicted by the OrthoMCL method from the PLAZA database (Van Bel et al., 2012) as the gene family and grouped 171 DNA repair genes of *A. thaliana* into 121 gene families.

**Table 1:** List of plant species in the dataset. 61 plant species including trees, perennial herbs, annual herbs, and algae were used for analyses. Two algae species (*Chlamydomonas reinhardtii* and *Micromonas commoda*) were eliminated from the analyses considering the phylogenetic relationships (PGLS analyses) because the no-sequence data of these species were available.

	Species name
Tree: 23 species	
Angiosperm	*Amborella trichopoda*
	*Carica papaya*
	*Citrus clementina*
	*Coffea canephora*
	*Eucalyptus grandis*
	*Hevea brasiliensis*
	*Malus domestica*
	*Populus trichocarpa*
	*Prunus persica*
	*Pyrus bretschneideri*
	*Theobroma cacao*
	*Ziziphus jujuba*
Gymnosperm	*Cycas micholitzii*
	*Ginkgo biloba*
	*Gnetum montanum*
	*Picea abies*
	*Picea glauca*
	*Picea sitchensis*
	*Pinus pinaster*
	*Pinus sylvestris*
	*Pinus taeda*
	*Pseudotsuga menziesii*
	*Taxus baccata*
Perennial herb: 15 species	
	*Arabidopsis lyrata*
	*Brassica oleracea*
	*Cajanus cajan*
	*Capsicum annuum*
	*Erythranthe guttata*
	*Fragaria vesca*
	*Marchantia polymorpha*
	*Nelumbo nucifera*
	*Oryza sativa* ssp. *japonica*
	*Ricinus communis*
	*Selaginella moellendorffii*
	*Solanum lycopersicum*
	*Solanum tuberosum*
	*Trifolium pratense*
	*Utricularia gibba*
Annual herb: 21 species	
	*Amaranthus hypochondriacus*
	*Arabidopsis thaliana*
	*Arachis ipaensis*
	*Beta vulgaris*
	*Brassica rapa*
	*Capsella rubella*
	*Chenopodium quinoa*
	*Cicer arietinum*
	*Citrullus lanatus*
	*Corchorus olitorius*
	*Cucumis melo*
	*Cucumis sativus*L.
	*Daucus carota*
	*Glycine max*
	*Medicago truncatula*
	*Petunia axillaris*
	*Physcomitrella patens*
	*Schrenkiella parvula*
	*Tarenaya hassleriana*
	*Vigna radiata* var*. radiata*
	*Zea mays*
Alga: 2 species	
	*Chlamydomonas reinhardtii*
	*Micromonas commoda*

**Figure 1:**
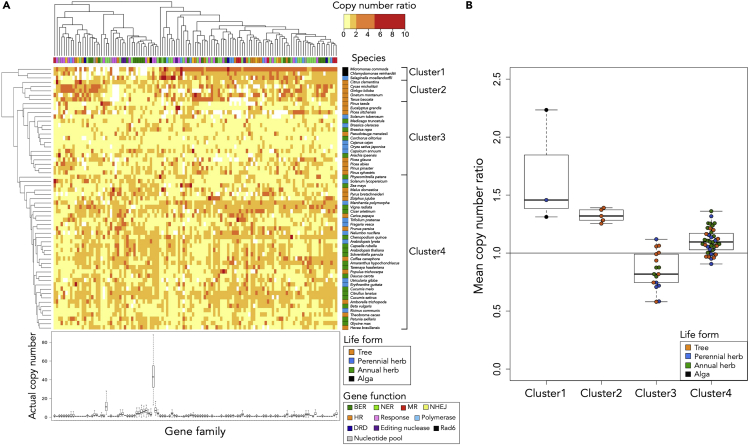
Interspecies comparison of copy number ratio of 121 DNA repair gene families (A) Clustered heatmap of the copy number ratio of 121 DNA repair gene families. Hierarchical clustering was performed based on the Euclidean distance of the copy number ratio of each species using the Ward’s method. 23 tree species, 15 perennial herb species, 21 annual herb species, and two algae species were included, and the life form of each species was in colored. Each gene family was categorized into one of 11 groups, and the function of each gene family was in colored: BER, base excision repair; NER, nucleotide excision repair; MR, mismatch repair; NHEJ, non-homologous end-joining repair; HR, homologous recombination repair; Response, DNA damage response; Polymerase, DNA polymerase; DRD, direct reversal of damage; Editing nuclease, editing and processing nuclease; Rad6, Rad6 pathway; Nucleotide pool, modulation of nucleotide pool. The actual copy number within each gene family is shown at the bottom of the figure. (B) Mean copy number ratios of 121 DNA repair gene families of species in the cluster. The horizontal line inside the box showed the median and the length of box showed the interquartile range (range between the 25th to 75th percentiles). The whiskers indicated points within 1.5 times the interquartile rage. The colors of the points correspond to the life form of the species.

Hierarchical clustering based on the similarity of the copy number ratio between species showed that 61 species were divided into four clusters (Figure 1A). Cluster1 consisted of three species, which were two algae species and one perennial herb (lycophyte) species, revealing significant enrichment of algae species (Fisher’s exact test; Q-value = 0.0262) (Table S2). The average of the mean copy number ratio over 121 DNA repair gene families was higher, but not significantly different from the mean of all species and other clusters (*t*-test; Q-value = 0.145) (Figure 1B). Cluster2 consisted of only five species, all of which were trees (one angiosperm and four gymnosperms), revealing significant enrichment of tree species (Fisher’s exact test; Q-value = 0.0452) (Table S2). The average of the mean copy number ratio in Cluster2 was significantly larger than the mean of all species and other clusters (*t*-test; t-value = 12.55, P-value = 2.32 × 10^−4^, Q-value = 4.64 × 10^−4^) (Figure 1B). In Cluster3, which consisted of 17 species, the average of the mean copy number ratio was significantly lower than the mean of all species (*t*-test; t-value = −3.83, P-value = 0.00147, Q-value = 0.00197) (Figure 1B). Cluster3 included eight tree, five perennial herb, and four annual herb species, revealing no significant enrichment or dilution of a certain type of life form (Table S2). Cluster4 included the largest number of species, in which the average of the mean copy number ratio was significantly larger than the mean of all species (*t*-test; t-value = 5.80, P-value = 1.42 × 10^−6^, Q-value = 5.69 × 10^−6^) (Figure 1B). Among the 36 species in Cluster4, ten species were trees, nine were perennial herbs, and 17 species were annual herbs. There was no significant enrichment or dilution of a certain type of life form (Table S2).

An alga, *Micromonas commoda*, is a unique species with low similarity of copy number ratio compared to the other species studied here (Figure 1A). In *M. commoda*, the copy number ratio was greater than the mean of all species in 105 gene families, whereas it was zero in 16 gene families (Figure 1A). Such a clear contrast of high and low copy number ratios among gene families was also found in another alga species, *Chlamydomonas reinhardtii*, and gymnosperm tree species, such as *Ginkgo biloba* and *Picea sitchensis*, but the pattern of the gene families with a high copy number ratio or a zero copy number ratio varied among species. This result suggests that each gene family has a species-specific history of gene loss and gene duplication.

The mean of actual copy number over species in each gene family was smaller than five and variance among species was low in most of the gene families (Figure 1A). However, in several gene families, the mean and variance of actual copy number was extremely large. For example, in the gene family involved in protein kinase production, including checkpoint kinase 2 (*CHEK2*), which participates in the DNA damage response in many cell types (Cybulski et al., 2004), and the cullin family, including cullin 4 (*CLU4*), which is involved in repair of UV-induced DNA lesions (Molinier et al., 2008), the means of the actual copy number were 47.07 and 15.61, and the variances of the actual copy number were 499.04 and 513.52, respectively (Figures 1A and S1). The phylogenetic signals in these gene families that had large mean copy numbers and large variance among species were weak (e.g., the estimated Pagel’s lambda in the protein kinase gene family was 7.55 × 10^−5^; and 0.077 in the cullin family). In addition, there was no significant relationship between the copy number and the life forms. Conversely, these gene families showed a positive correlation between the copy number and the total number of genes in a species (e.g., Spearman’s rank correlation coefficient in the protein kinase gene family was 0.77; and 0.61 in the cullin family). This suggests that the family sizes of protein kinase and cullin increased with the genome size expansion.

### Extracting the DNA repair gene family that showed a high copy number ratio in tree species

Next, we investigated whether copy number ratios are significantly different among tree, perennial, and annual herb species for each gene family using phylogenetic generalized least squares (PGLS). The phylogenetic signals in the copy number ratio varied depending on the gene family (Table S3). The estimated values of Pagel’s lambdas were smaller than 0.1 in 60 gene families (e.g., poly(ADP-ribose) polymerase [*PARP*], breast cancer 2 [*BRCA2*], and DNA damage-binding protein [*DDB*]), and were greater than 0.1 in 61 gene families (e.g., DNA glycosylase superfamily protein [*Tag*], replication protein A2 [*RPA2*] and structural maintenance of chromosomes 6 [*SMC6*]) (Table S3).

**Figure 2:**
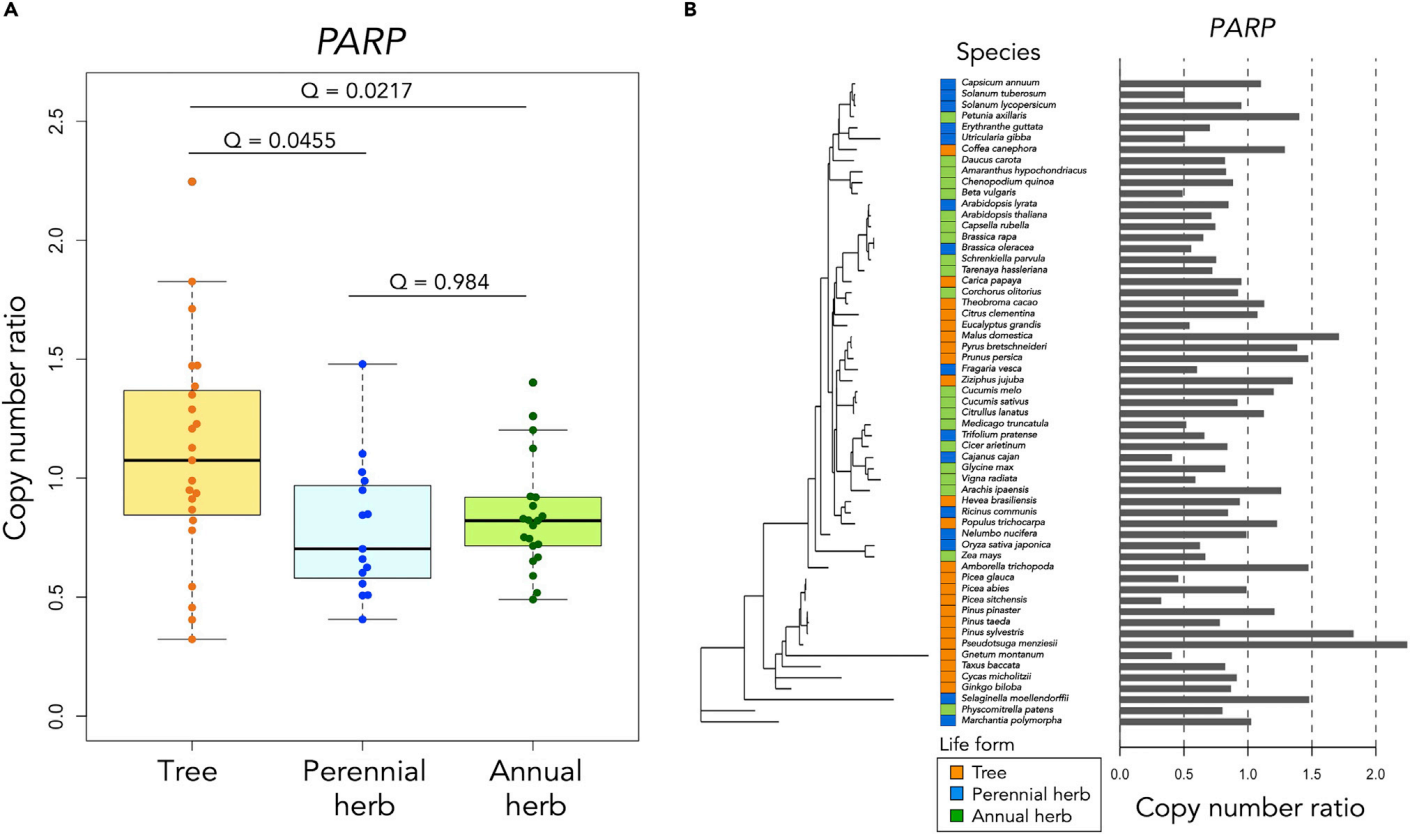
The result of phylogenetic generalized least squares regressions (A) The copy number ratio of *PARP* in each life form. The result of the PGLS regressions showed that tree species had significantly higher copy number ratios in the *PARP* gene family compared to perennial herb species (coefficient = −0.395, standard error = 0.111, *t*-value = −3.560, P-value = 7.659 × 10^−4^, Q-value = 0.0455) and annual herb species (coefficient = −0.363, standard error = 0.090, *t*-value = −4.014, P-value = 1.794 × 10^−4^, Q-value = 0.0217). The horizontal line inside the box showed the median and the length of box showed the interquartile range (range between the 25th to 75th percentiles). The whiskers indicated points within 1.5 times the interquartile rage. The points beyond the whisker range indicated the outliers. (B) The phylogenetic relationships in the copy number ratio of *PARP*. The estimated Pagel’s lambda was 4.97 × 10^−9^.

Among the 121 gene families, only one showed a significantly higher copy number ratio in tree species than in perennial and annual herb species, which was poly(ADP-ribose) polymerase (*PARP*s) (Figure 2A). Another gene family (*Tag*) showed a significantly higher copy number ratio in tree species than in perennial herb species, but the difference between tree and annual herb species was not significant in this gene family (Figure S2). The three species with the highest copy number ratio of *PARP*s were *Pseudotsuga menziesii* (Douglas-fir), *Pinus sylvestris* (Scots pine), and *Malus domestica* (apple) (Figure 2B). Douglas-fir and Scots pine are known as long-lived conifers and can live for over 1000 years (Franklin and Dyrness, 1973). Apple trees live between 60 and 100 years (Pereira-Lorenzo et al., 2009). Although the longevity of the apple tree is not as long as that of conifers, it is significantly longer than that of herb species.

PARPs are key enzymes associated with poly(ADP-ribosyl)ation. Poly(ADP-ribosyl)ation is a covalent post-translational modification process of proteins via the synthesis and transfer of poly ADP-ribose from NAD^+^ to target proteins (Rissel and Peiter, 2019). The ADP-ribose polymer formed by the sequential attachment of ADP-ribosyl moieties attracts enzymes for DNA repair, particularly those associated with BER and other types of ssDNA repair. *PARP*s are found in all eukaryotic supergroups (Citarelli et al., 2010) and *A. thaliana* encodes three canonical PARP proteins (AtPARP1, AtPARP2, and AtPARP3).

Poly(ADP-ribosyl)ation is reversible and the covalently attached poly(ADP-ribose) from acceptor proteins are removed by poly(ADP-ribose) glycohydrolase (PARG) enzymes (Briggs and Bent, 2011; Vainonen et al., 2016). PARP and PARG proteins interact with each other, and the cellular pools of ADP-ribose are regulated. Because plant PARGs are also involved in DNA repair and biotic/abiotic stress responses (Li et al., 2011; Zhang et al., 2015; Song et al., 2015), we compared the copy number ratio of *PARG* genes among lifeforms. We found there was no significant difference in the copy number ratio of *PARG* gene family between tree species and perennial herb species (Q-value was 0.732) and between tree species and annual herb species (Q-value was 0.286), although the copy number ratio in tree species was lower than those in herb species. This result suggests that increased copy number of *PARG*s is not essential for DNA repair and the longevity in plants.

### The *PARP* gene family was divided into three functional groups

**Figure 3:**
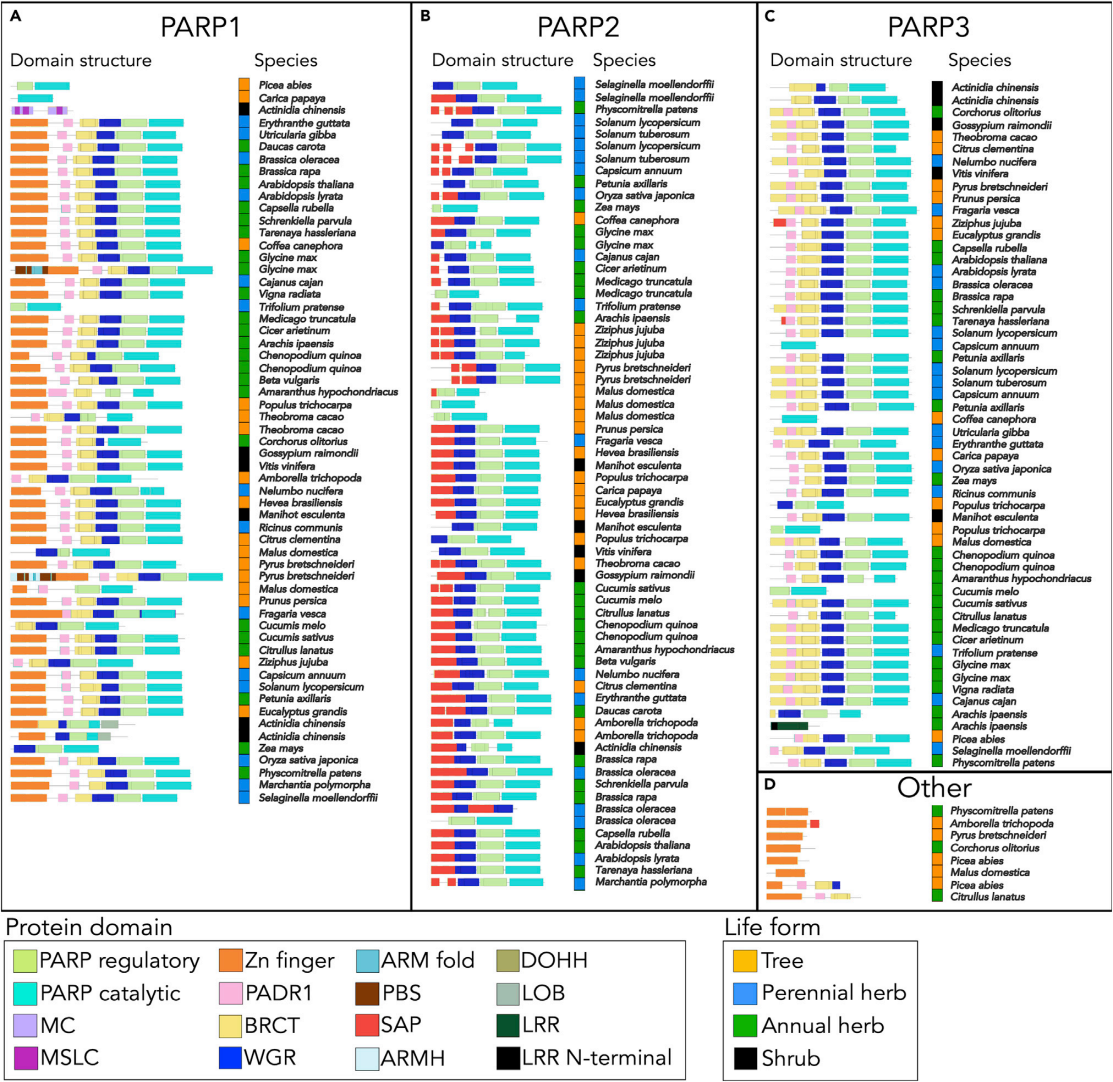
The protein domain structures of PARPs of species in Dicots PLAZA 4.0 dataset Each PARP was categorized into four groups (A) PARP1, (B) PARP2, (C) PARP3 and (D) Other based on the annotations in Dicots PLAZA 4.0 and the phylogenetic tree constructed by the tree explore tool in Dicots PLAZA 4.0. Protein domains are illustrated by colored. PARP regulatory: Poly(ADP-ribose) polymerase regulatory domain, PARP catalytic: Poly(ADP-ribose) polymerase catalytic domain, MC: Mitochondrial carrier domain, MSLC: Mitochondrial substrate/solute carrier domain, Zn finger: zinc-finger domain, PADR1: PADR1 domain, BRCT: BRCA1 C terminus domain, WGR: tryptophan-glycine–arginine-rich domain, ARM fold: Armadillo-type fold domain, PBS: PBS lyase HEAT-like repeat domain, SAP: SAF-A/B, Acinus and PIAS domain, ARMH: Armadillo-like helical domain, DOHH: Deoxyhypusine hydroxylase domain, LOB: Lateral organ boundaries domain, LRR: Leucine-rich repeat domain, LRR N-terminal: Leucine-rich repeat-containing N-terminal domain.

189 *PARP* genes in dicot species were divided into four distinct clades based on sequences and protein domain structures using the tree explore tool in Dicots PLAZA 4.0 (Figure 3). One clade consisted of 59 genes from 52 species and was named as the *PARP1* clade because almost all members were characterized by a highly conserved domain structure of *Arabidopsis PARP1* (Figure 3A). *Arabidopsis PARP1* possesses an N-terminal DNA interaction domain (Zinc-finger), a C-terminal catalytic domain (PARP catalytic; Rissel and Peiter, 2019), a PARP regulatory domain (PARP regulatory), and a WGR domain, named after its repeating amino acid motif (W-G-R), located in the central region. The *PARP2* clade consisted of 66 genes in 48 species, including *Arabidopsis PARP2* (Figure 3B). Almost all members of the *PARP2* clade lack the zinc-finger domains but possess SAF-A/B, acinus, and PIAS (SAP) domains in the N-terminus, consistent with the previous characterization of *Arabidopsis* PARP2 (Lamb et al., 2012). The SAP domain has been shown to bind to nucleic acids (Okubo et al., 2004), suggesting the ability of DNA binding for PARP2 protein. Another clade, named as the *PARP3* clade, consisted of 56 genes in 48 species, including *Arabidopsis PARP3* (Figure 3C). The domain structure of the *PARP3* clade members resembles those of the *PARP1* clade, but members of the *PARP3* clade lack the zinc-finger domains, consistent with the finding of previous study based on *A. thaliana* (Vainonen et al., 2016).

Members in a minor clade (named “Other”), consisted of eight genes and had only zinc-finger domains, implying no catalytic or regulatory functions (Figure 3D). BLAST search against human genome showed that the sequences of these genes are the most similar to human *PARP1* gene rather than other human *PARP* genes. In addition, the sequences of these genes were more similar to plant *PARP2* gene rather than radical-induced cell death 1 (*RCD1*) gene and Similar to RCD one (*SRO*s) genes, which encode proteins containing PARP-like domains (Jaspers et al., 2010).

The phylogenetic tree constructed from the plant species, including angiosperms, gymnosperms, lycophytes, and bryophytes, also showed that plant *PARP* genes were divided into three distinct clades of *PARP1*, *PARP2*, and *PARP3* (Figure S3), suggesting that three paralogs of the *PARP* genes were present in the common ancestor of angiosperms, gymnosperms, lycophytes, and bryophytes.

### Tree species have higher copy number ratios in *PARP1* and *PARP2* but not in *PARP3*

**Figure 4:**
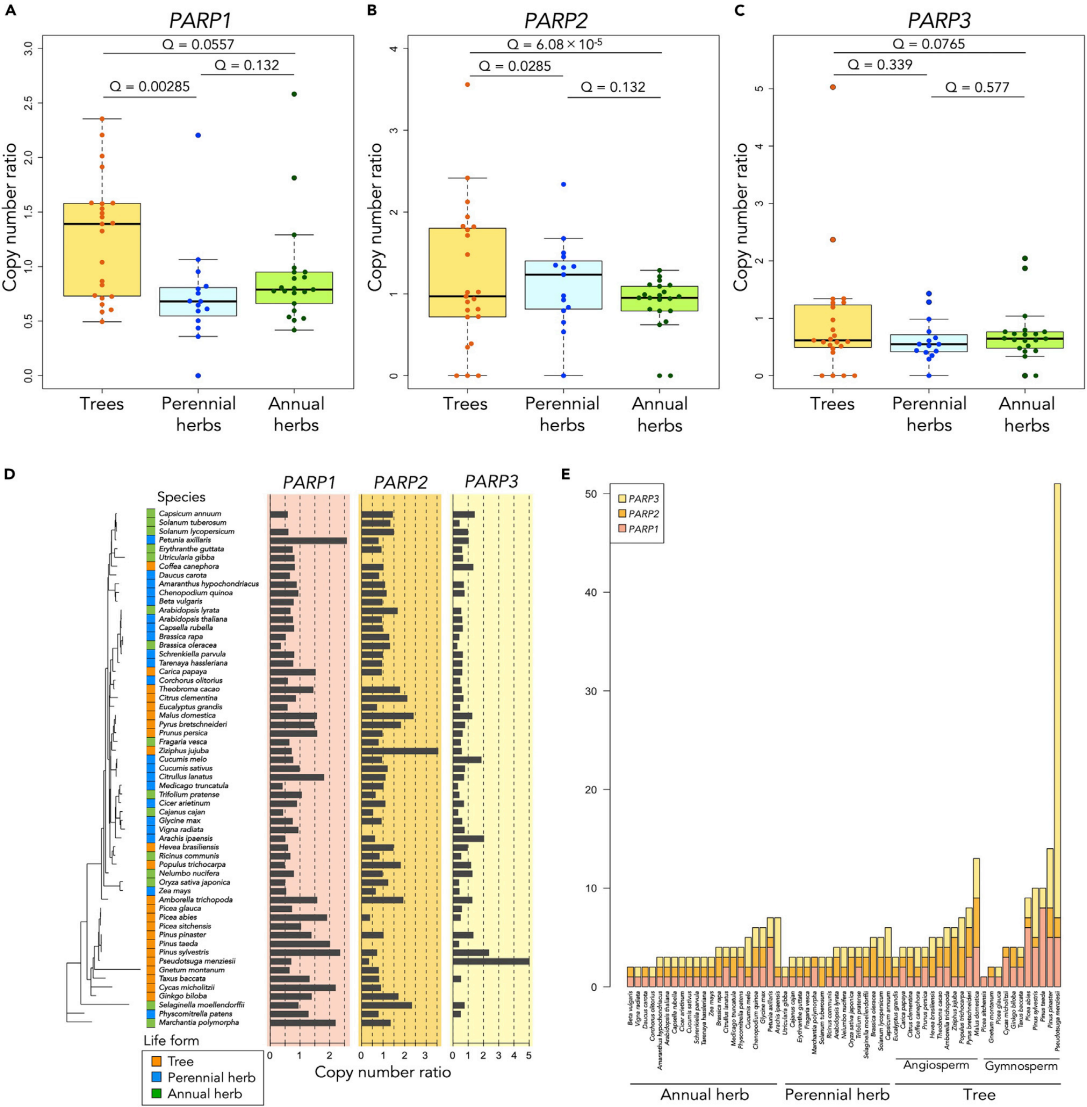
Comparison analyses for each type of *PARP* (A–C) Comparison of copy number ratios in *PARP1* (A), *PARP2* (B) and *PARP3* (C) among life forms by PGLS regressions. Tree species had significantly higher copy number ratio than perennial herb species and annual herb species in *PARP1*. Also, tree species had significantly higher copy number ratios in *PARP2* than perennial herb species and annual herb species. The copy number ratios of *PARP3* in tree species were not significantly different compared to perennial herb species and annual herb species. The horizontal line inside the box showed the median and the length of box showed the interquartile range (range between the 25th to 75th percentiles). The whiskers indicated points within 1.5 times the interquartile rage. The points beyond the whisker range indicated the outliers. (D) The phylogenetic relationships of copy number ratios in *PARP1*, *PARP2*, and *PARP3*. (E) The actual copy number of *PARP* genes in the species.

The copy number ratios of the members of *PARP1* and *PARP2* clades were significantly higher in tree species than in annual and perennial herb species (Figures 4A and 4B and Table 2), but there was no significant difference between life forms for *PARP3* (Figure 4C and Table 2). The tree species that showed the highest copy number ratio of each *PARP* gene were different: *Pinus sylvestris*, *Ziziphus jujuba*, and *Pseudotsuga* *menziesii* showed the highest copy number ratios of *PARP1*, *PARP2*, and *PARP3*, respectively (Figure 4D).

**Table 2:** The result of PGLS regressions to compare the copy number ratios among life forms for each paralog of *PARP* gene family.

	Trees versus annual herbs				Trees versus perennial herbs				
Gene	Coefficient	Standard error	*t*-value	Q-value	Coefficient	Standard error	*t*-value	Q-value	Pagel's lambda
*PARP1*	−0.280	0.131	−2.135	0.0557	−0.541	0.161	−3.361	0.00285	7.54 × 10^−9^
*PARP2*	−0.973	0.209	−4.655	6.1 × 10^−5^	−0.686	0.21	−3.263	0.00285	0.878
*PARP3*	−0.316	0.175	−1.80	0.0765	−0.2.07	0.215	−0.964	0.339	8.04 × 10^−9^

The actual copy number of *PARP* genes was also large in tree species, especially in gymnosperms (*P. sylvestris*, *Pinus taeda*, *Pinus pinaster*, and *P. menziesii*: Figure 4E). *P. taeda* had eight *PARP1* genes, the largest number of *PARP1* genes among all species. *P. menziesii* had 44 *PARP3* genes, the largest number of *PARP3* genes among all species. All tree species had at least one *PARP1* gene, but some gymnosperms had lost the *PARP2* and/or *PARP3* genes (Figure 4E), suggesting that *PARP1* is the most essential gene for long-lived trees.

### An inverse relationship between copy number ratios in *PARP*s and growth rate in tree species

Next, we tested whether there is a significant association between copy number ratio of *PARP*s and longevity. Because reliable estimation of plant lifespan is difficult and maximum tree lifespans published in prestigious scientific journals are not always supported by scientific evidence (Piovesan and Biondi, 2020), we used growth rate (the rate of height increment) instead of lifespan. In the field of forest ecology, there is a longstanding argument that slow-growing trees live longer than fast-growing trees (Johnson and Abrams, 2009; Black et al., 2008). Because the data for growth rate can be more easily available than those for longevity, we collected the growth data in 11 tree species including angiosperms and gymnosperms from previous studies (Köstler, 1956; Burns and Honkala, 1990a; 1990b; Liebhard et al., 2003; Bravo-Oviedo et al., 2004) (Table S4) and investigated the relationship between the growth rate and the copy number ratio of *PARP*s using phylogenetic generalized least squares (PGLS) regression analyses. Because inverse relationship between growth rate and longevity has been argued mainly in tree species, and height growth rate is difficult to obtain in herbs, we applied this analysis only for tree species.

**Figure 5:**
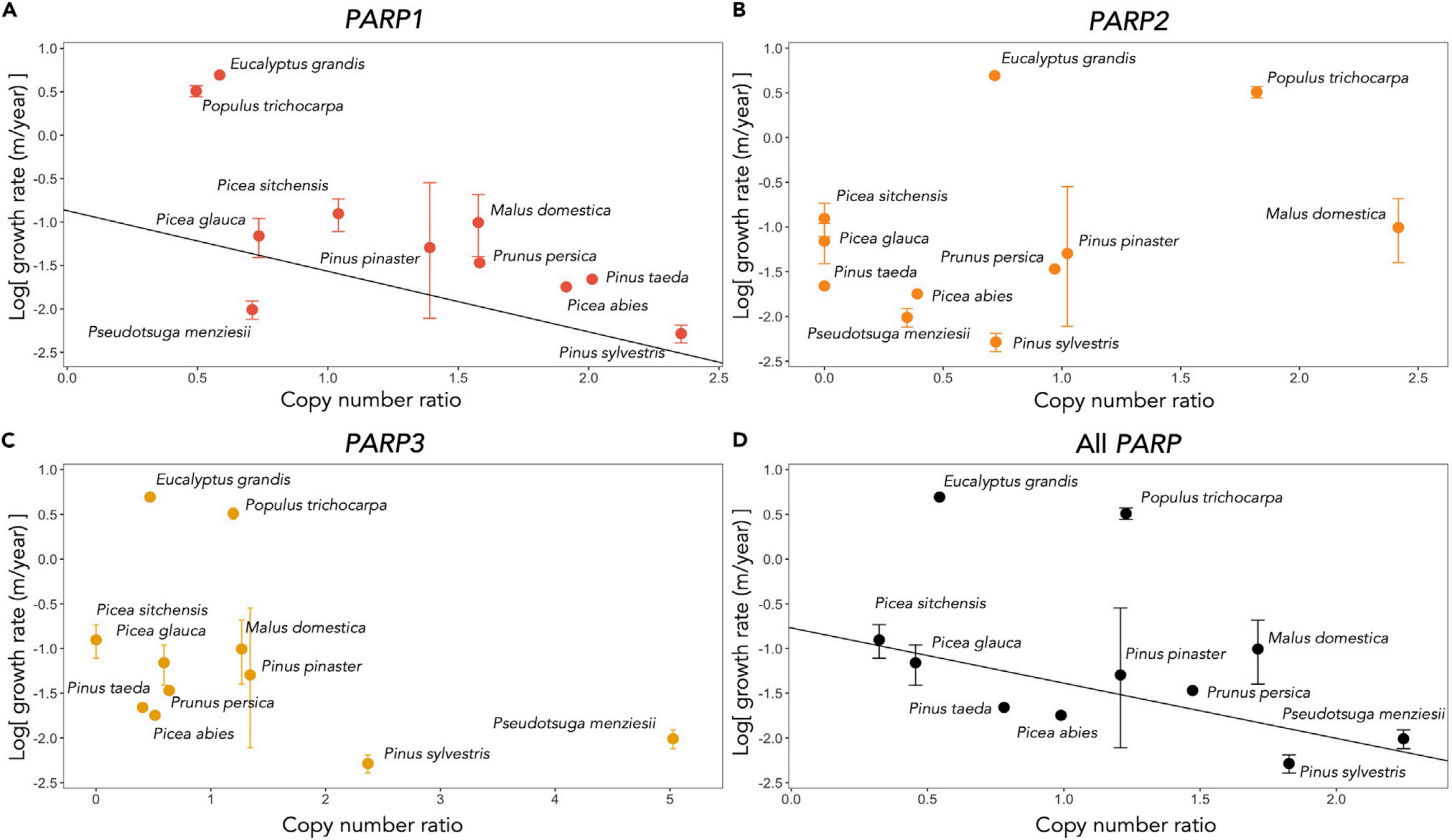
The relationships between growth rate and copy number ratio of each *PARP* in 11 tree species Plots showed the average height growth rate (m/year) and vertical bar showed the highest and lowest growth rate of the species. There were significantly negative correlations between the copy number ratio and log growth rate in *PARP1* (Q-value was 0.0173 in PGLS) (A) and all type of *PARP* including *PARP1*, *PARP2*, and *PARP3* (Q-value was 0.00601 in PGLS) (D). There were no significant relationships between the copy number ratio and log growth rate in *PARP2* (B) and *PARP3* (C).

There was significantly negative correlation between log growth rate (m/year) and the copy number ratio in *PARP* gene family (Figure 5) (Table 3). Among three *PARP* family members, the significantly negative correlation between log growth rate and the copy number ratio was shown only in *PARP1* (Figure 5) (Table 3). This result strongly suggests the important role of PAPR1 for slow growth and longevity in tree species.

**Table 3:** The result of regressions to investigate the relationships between growth rate and copy number ratio of *PARP*s in 11 tree species by phylogenetic generalized least squares (PGLS) regressions.

	Coefficient	Standard error	*t*-value	Q-value	Pagel's lambda
*PARP1*	−0.698	0.194	−3.599	0.0173	1
*PARP2*	−0.231	0.441	−0.524	0.613	0.323
*PARP3*	−0.181	0.0770	−2.349	0.0651	0.110
All *PARP*	−0.618	0.173	−3.571	0.00601	0.399

## Discussion

To examine the role of DNA repair in plant longevity, we systematically compared the copy number variations of 121 DNA repair gene families in 61 plant species, including trees, annual/perennial herbs, and algae. Among the diverse DNA repair gene families studied here, the *PARP* gene family was identified as the only one that revealed significant expansion in tree species relative to annual/perennial herb species. The long-lived conifers, Douglas-fir and Scots pine, as well as fruit tree (apple tree) were found to be the species with highest copy number ratios of *PARP*s. These results suggest that selection probably promotes convergent evolution of increased copy numbers of *PARP*s in tree species.

As key enzymes associated with poly(ADP-ribosyl)ation, PARPs have been extensively studied in animals. The *PARP* gene family is considerably larger in vertebrates than in plants. In humans, there are 17 family members that share the PARP catalytic domain of PARP1 (Amé et al., 2004; Hottiger et al., 2010). Our analyses showed that 59 plant species, including angiosperms, gymnosperms, lycophytes, and bryophytes have only two or three *PARP* family members (Figures 3 and 4E). PARP proteins in *A. thaliana* (AtPARP1, 2, and 3), *Zea mays* (maize) and *Glycine max* (soybean) have confirmed or predicted poly ADP-ribosylation activity (Jaspers et al., 2010; Babiychuk et al., 1998; Amor et al., 1998), and AtPARP1 and AtPARP3 are structurally the most similar to human PARP1, whereas AtPARP2 is similar to human PARP2, indicating the functional similarities between *Arabidopsis* and human PARPs (Rissel and Peiter, 2019).

Among three *PARP* family members in plants, only the copy number ratios of the two members, *PARP1* and *PARP2*, were significantly higher in tree species than those in annual and perennial herb species (Figure 4). In *A. thaliana*, AtPARP1 and AtPARP2 play the predominant role in poly(ADP-ribose) polymerase activity and DNA damage response (Song et al., 2015; Gu et al., 2019). In contrast to *AtPARP1* and *AtPARP2*, the expression of *AtPARP3* is restricted to seed tissues (Rissel et al., 2014). Moreover, a recent study reported that AtPARP3 does not have poly(ADP-ribose) polymerase activity (Gu et al., 2019). Together with these previous reports, our results suggest that increased copy numbers of *PARP*s that are capable of adding ADP-ribose units onto protein substrates are likely to be evolutionary favored in long-lived tree.

The best-studied PARPs, including the founding member PARP1, catalyze the formation of long, branched chains of ADP-ribose, known as poly (ADP-ribose) (PAR) (Hassa and Hottiger, 2008; Gibson and Kraus, 2012). These PAR-forming enzymes perform functions such as nucleation of DNA-damage foci (PARP1 and 2) and proper chromosome segregation during mitosis (PARP5a in human) (Schreiber et al., 2006; Hassa and Hottiger, 2008). Although historically PARP1 in animals has been studied with the focus on DNA damage detection and repair, more recently it has been understood that in the absence of DNA damage, PARP1 also plays an important role in regulating chromatin structure and gene expression by biding near the promoters of transcriptionally active genes (Krishnakumar and Kraus, 2010). Cell survival after genotoxic stress is determined by a counterbalance of pro- and anti-death factors. Sirtuins (SIRTs) are deacetylases that promote cell survival, whereas poly(ADP-ribose) polymerases (PARPs) can act both as survival and death inducing factor. The two protein families are strictly dependent on the oxidized form of nicotinamide adenine dinucleotide (NAD^+^) for their activities. Previous studies have reported that increased activity of PARP1, but not overexpression, is associated with longevity of mammalian species (Grube and Bürkle, 1992). Furthermore, increased amounts sirtuins are associated with improved health and longevity in mammals (Mouchiroud et al., 2013). Although less is known about the functions of plant PARPs in contrast to their mammalian counterparts, AtPARP1 and AtPARP2 have been shown to be associated with DNA repair (Doucet-Chabeaud et al., 2001; De Block et al., 2005) and transcriptional regulation (Babiychuk et al., 2001; Storozhenko et al., 2001; Vanderauwera et al., 2007). Our findings that long-lived trees have higher copy number ratio of *PARP*s than herbs will lead to the intriguing hypothesis that PARPs play an important role on aging and longevity both in plants and animals.

The pharmacological and genetic inhibition of PARP in *A. thaliana* results in an increased stress tolerance and increased growth by preventing cell death (De Block et al., 2005) but it also leads to reduced defense because of the reduced accumulation of protective molecules, especially anthocyanin and ascorbate (Schulz et al., 2012). The antagonistic relationship between increased growth and decreased defense by inhibition of PARP provides an important insight into the long-standing ecological argument that slow-growing trees live longer than fast-growing trees (Johnson and Abrams, 2009; Black et al., 2008). Long-lived, late successional species typically grow more slowly, invest more resources for defensive compounds and structural support, and maintain lower rates of photosynthesis and respiration than shorter-lived, early successional species (Loehle, 1988). Although the underlying molecular mechanism for long-lived and short-lived tree species remained completely unknown, our finding provides the new testable hypothesis that increasing copy number of *PARP*s enhance allocation to defensive compounds that leads to slow growth and great longevity. Indeed, the plot of growth rate against the copy number ratio of *PARP1*s showed a significant negative correlation (Figure 5).

In mammals, there is a clear positive correlation between activity of PARPs and longevity (Grube and Bürkle, 1992), although the copy number of *PARP* genes are not so different among species with different life spans (MacRae et al., 2015a). Given these previous reports in mammals, we speculate that the enhanced activity of PARPs could contribute to the longevity in animals, while an increased copy number of *PARP*s is more likely to occur in long-lived plants. The difference between animals and plants may be originated from the different history of genome evolution. In plants, whole genome duplication and polyploidization events occurred more frequently than those in animals (Murat et al., 2012). Because frequent duplication and polyploidization would lead to dynamic and faster genome evolution, the copy number of *PARP*s could change more flexible in plants than in mammalian genomes that are conserved and stable.

Another important function of PARPs is to regulate viral infectivity and pathogenesis (Kuny and Sullivan, 2016). In humans, PARP13 has been reported to reveal broad antiviral activity through direct biding of viral RNA by PARP13, followed by recruitment of the exosome and specific degradation of viral RNA (Gao et al., 2002; Müller et al., 2007; Bick et al., 2003; Mao et al., 2013). Daugherty et al. (2014) demonstrated that nearly one-third of primate *PARP* genes, including *PARP13*, are evolving under strong recurrent positive selection, implicating the essential role of PARPs in antiviral defense in mammalian genomes. The role of PARPs in antipathogen defense can also be identified in plants. In *A. thaliana*, AtPARP2 has been demonstrated to regulate the response to pathogen infection and repair of pathogen-induced DNA damage (Song et al., 2015). Because long-lived trees are exposed to the continuous risk of pathogen-induced DNA damage, protection of the plant host genome against pathogen invasion is essential (Song and Bent, 2014). A recent comparative genomics study showed the clear expansion of plant resistance genes (R-genes) and orthologs related to plant immunity in trees relative to herbs (Tobias and Guest, 2014; Plomion et al., 2018). An increased copy number of *PARP*s could provide another mechanism of antipathogen defense that is necessary for the success of long-lived trees.

In addition to the *PARP* gene family, our hierarchical clustering analysis results (Figure 1) showed that the increased copy number ratio of various DNA repair gene families may contribute to the longevity of some tree species, including *Citrus clementina*, *Cycas micholitzii*, *Ginkgo biloba*, *Gnetum montanum*, and *Taxus baccata*. DNA damages varied from basal lesions to DNA double-strand breaks (DSBs) due to various genotoxic stresses, and such DNA damages can be repaired by various DNA repair. Previous studies showed the positive correlation between the activities of DNA repair in multiple pathway and longevity in animal species. Humans and naked mole-rats, which have long lifespans, have significantly higher expression levels of DNA repair genes including genes involved in DNA damage sensing, mismatch repair (MMR), non-homologous end-joining (NHEJ) repair and base excision repair (BER) than mouse (MacRae et al., 2015b). DNA repair genes involved in BER and repair of DNA DSBs are more highly expressed in long-lived bat species than in short-lived bat species (Huang et al., 2020). Thus, the coevolution of copy number variations of DNA repair genes in multiple pathways may provide a strategy for efficient DNA repair, contributing to the success of long-lived organisms. Comparison of expression profiles of DNA repair genes including *PARP*s among plant species with different lifespans will be extremely interesting in future studies.

Among 121 DNA repair gene families studied, only one gene family, *PARP* gene family, was identified as the gene family that revealed significant expansion in tree species relative to annual and perennial herb species. Although some gene families also had an important role in DNA repair, significant expansion in tree species relative to herb species was not found in most gene families. This is because the number of species in the dataset was not large enough and species were limited. In spite of the limitation of data, *PARP* gene family was found to have significantly higher copy number ratio in tree species than annual and perennial herb species. This suggests that *PARP* gene family is a strong candidate gene family associated with tree longevity.

Overall, systematic comparative analyses of the copy number variations in DNA repair genes in diverse species demonstrates that *PARP*s, especially *PARP1* and *PARP2*, are strong candidate genes associated with tree longevity. PARPs have pivotal roles in the response to and repair of DNA damage, including basal and bulky lesions and single- and double-strand breaks due to endogenous and exogenous stresses. The result of our study can be a foundation for researches to elucidate the relationships of DNA repair and the evolution of species longevity in plants. As genome sequences of more diverse plant species become available, systematic comparative genome analyses will provide important clues to reveal the relationships of DNA repair and the evolution of longevity in diverse organisms.

### Limitations of the study

We collected the information regarding the copy number of DNA repair genes in plant species from the PLAZA database, the genomic database of diverse plant species. We used Dicots PLAZA 4.0 and Gymno PLAZA 1.0 so that we could cover both angiosperms and gymnosperms. The predicted copy number of DNA repair genes are largely derived from newly sequenced plant genomes using homologous sequences. The estimates may therefore not accurately represent true biological gene numbers and should be interpreted with caution. We also acknowledge that 61 species used for our analyses may not be sufficient. Thanks to the advances in DNA sequencing technology, genomes from increasingly large number of species will be available in the near future. Applying our analyses to the larger set of data will uncover new DNA gene families that could be involved in tree longevity.

## STAR★Methods

### Key resources table

**Table:** 

REAGENT or RESOURCE	SOURCE	IDENTIFIER
**Software and algorithms**		

R Version 3.6.3		http://www.r-project.org/
MEGA X version 10.1.5	Kumar et al. (2018)	https://www.megasoftware.net/
RaXML version 8.0.0	Stamatakis (2014)	https://cme.h-its.org/exelixis/web/software/raxml/
NCBI Taxonomy Browser		https://www.ncbi.nlm.nih.gov/Taxonomy/Browser/wwwtax.cgi
Tree explore tool in Dicots PLAZA 4.0		https://bioinformatics.psb.ugent.be/plaza/versions/plaza_v4_dicots/
MaxAlign	Gouveia-Oliveira et al. (2007)	http://www.cbs.dtu.dk/services/MaxAlign/
MAFFT version 7	Katoh et al. (2018)	https://mafft.cbrc.jp/alignment/software/
BLAST+	Camacho et al. (2009)	https://blast.ncbi.nlm.nih.gov/Blast.cgi?PAGE_TYPE=BlastDocs&DOC_TYPE=Download
qvalue package in R version 2.16.0	Storey et al. (2015)	http://github.com/jdstorey/qvalue
phylolm package in R version 2.6	Tung Ho and Ané (2014)	https://CRAN.R-project.org/package=phylolm

**Other**		

Copy number data of 121 gene families in 61 plant species from Gymno PLAZA 1.0 and Dicots PLAZA 4.0	Gymno PLAZA 1.0; Proost et al. (2009)	https://bioinformatics.psb.ugent.be/plaza/versions/gymno-plaza/
	Dicots PLAZA 4.0; Van Bel et al. (2018)	https://bioinformatics.psb.ugent.be/plaza/versions/plaza_v4_dicots/
Sequence data of *rbcL* and *matK* to construction of phylogenetic tree from NCBI	National Center for Biotechnology Information (NCBI)	https://www.ncbi.nlm.nih.gov/
Data regarding the individual ages and heights in 11 tree species to calculate the growth rates	Köstler (1956); Burns and Honkala (1990a)and 1990b; Liebhard et al. (2003); Bravo-Oviedo et al. (2004)	N/A
The database used to categorize each species included in the database into five groups according to life form	PLANTS Database	http://plants.usda.gov/
	Plants of the World Online	http://www.plantsoftheworldonline.org/
	Plants For A Future	https://pfaf.org/user/Default.aspx
	The University and Jepson Herbaria	https://ucjeps.berkeley.edu/
	The University of Massachusetts Weed Herbarium	http://extension.umass.edu/landscape/weed-herbarium
	The Angiosperm Phylogeny Website	http://www.mobot.org/MOBOT/research/APweb/
	The Gymnosperm Database	https://www.conifers.org/

### Resouce availability

#### Lead contact

Further information and requests for resources and reagents should be directed to and will be fulfilled by the Lead Contact, Akiko Satake (akiko.satake@kyudai.jp).

#### Materials availability

This study did not generate new unique reagents.

#### Data and code availability

This paper analyses existing, publicly available data. The source of data for analyses is listed in the key resources table. This paper does not report original code. Any additional information required to reanalyze the data reported in this paper is available from the lead contact upon request.

### Experimental model and subject details

To collect the information regarding copy number of DNA repair genes in plant species, we used the PLAZA database, the genomic database of diverse plant species. We used Dicots PLAZA 4.0 (Van Bel et al., 2018) and Gymno PLAZA 1.0 (Proost et al., 2009) in order to cover both angiosperms and gymnosperms. These databases also include bryophytes (*Marchantia polymorpha* and *Physcomitrella patens*) and algae (*Chlamydomonas reinhardtii* and *Micromonas commoda*). We categorized each species included in the database into five groups according to life form: alga, annual herb, perennial herb, shrub, and tree based on the information from the databases (the PLANTS Database, Plants of the World Online, Plants For A Future, the University and Jepson Herbaria, the University of Massachusetts Weed Herbarium, the Angiosperm Phylogeny Website, and the Gymnosperm Database) and in the literature (Takasaki et al., 1994; Gotmare et al., 2000; Inan et al., 2004; Zhang et al., 2013b; Tivoli et al., 2006; Merchant et al., 2007; van Baren et al., 2016; Cove, 2005). The species name and number of species of each life form are listed in Table S5. We eliminated four shrub species (*Actinidia chinensis*, *Gossypium raimondii*, *Manihot esculenta*, and *Vitis vinifera*) from the analyses of life form comparison due to their intermediate life forms, which are tree-like, small sized (< 5 m), and have a relatively short lifespan. Thus, 61 species, including 23 tree species, 15 perennial herb species, 21 annual herb species, and two algae species were used for our analyses (Table 1).

### Methods details

#### Selecting genes associated with DNA repair

From the Dicots PLAZA 4.0 and Gymno 1.0 PLAZA databases, we selected 171 genes associated with DNA repair systems within *Arabidopsis thaliana* and categorized these genes into 11 functional groups depending on the pathways for DNA repair following Singh et al. (2010) (Table S1). We used the orthologous groups predicted by the OrthoMCL method from the PLAZA database (Van Bel et al., 2012) as the gene families and grouped 171 DNA repair genes of *A. thaliana* into 121 gene families.

#### The index of the copy number of genes for analyses

To compare the copy number of DNA repair genes between species, we needed to normalize the copy number of genes within each gene family in the focal species by the total number of genes in the species because the species with a large total number of genes would have a large number of DNA repair genes. The PLAZA database provided the copy number ratio rather than the actual copy number. The copy number ratio of the gene family *j* in species *i* was calculated based on four values: The sum of genes included in gene family *j* over all species (*N*_*j*_), the total number of genes included in gene family *j* in species *i* (*L*_*ij*_), the sum of the total number of genes over all species (*N*_*total*_), and the total number of gene in species *i* (*L*_*total,i*_). Using these four values, the copy number ratio is given as follows:

The copy number ratio of gene family *j* in species *i*= $\frac{L_{ij}}{L_{total,\;i}}/\frac{N_{j}}{N_{total}}$.

The numerator, $\frac{L_{ij}}{L_{total,\;i}}$, indicates the normalized copy number of the gene family *j* in species *i* that have total number of genes, *L*_*total,i*_. The denominator represents the fraction of the gene family *j* in the total number of genes. We can estimate whether the normalized copy number of the gene family *j* in species *i* is relatively higher compared to the average normalized copy number of the gene family *j* over all species of the dataset using this copy number ratio. By this normalization, the mean of the copy number ratio becomes one.

#### Construction of the phylogenetic tree

To adopt statistical methods to consider the phylogenetic relatedness of target traits, we first drew a phylogenetic tree using species included in database. We constructed a phylogenetic tree using the National Center for Biotechnology Information (NCBI) Taxonomy Browser (Figure S4). Then, to calculate the branch length of the phylogenetic tree, we collected the DNA sequences of the ribulose-1,5-bisphosphate carboxylase/oxygenase large subunit (*rbcL*) and maturaseK (*matK*) from the NCBI. Because the sequence data of *rbcL* of *Citrus clementina* was not found, the sequence of *rbcL* of *C. sinensis*, a closely related species of *C. clementina*, was used as an alternative. Because no sequence data for two algae species, *Chlamydomonas reinhardtii* and *Micromonas commoda*, was available, we eliminated these two algae species from the analysis. Thus, we used 59 species for the analyses that considered the phylogenetic relationships (Table S6). We aligned the sequences using the ClustalW algorithm in the program Molecular Evolutionary Genetics Analysis (MEGA) X (v. 10.1.5); Kumar et al., 2018). After alignment, we calculated the branch lengths of the phylogenetic tree using RAxML (v. 8.0.0; Stamatakis, 2014).

### Quantification and statistical analysis

#### The similarity of the copy number ratio between species

To assess the similarity of the copy number ratio between species, we performed hierarchical clustering based on the Euclidean distance of the copy number ratio of each species using the Ward’s method. To test the enrichment or dilution of each life form in each of the significantly different clusters, Fisher’s exact tests (two-sided) were performed. After the clustering, we tested whether the species in the cluster had a higher or lower copy number ratio than the mean of all species. The mean copy number ratio of 121 gene families within each species was calculated. Then, we tested whether the average of the mean copy number ratio of 121 gene families within species included in the cluster was significantly higher or lower than one (that is, mean copy number ratio of all species) by *t*-test. After the *t*-tests, to control for false discovery rate, we used the method of Storey’s Q-value (Storey, 2002), and the Q-value of each test was estimated using the q-value package (ver. 2.16.0); Storey et al. (2015) in R.

#### PGLS to investigate the relationship between the copy number ratio and life forms

Next, we explored the relationship between the copy number ratio and life forms in each gene family using phylogenetic generalized least squares (PGLS) regression (Grafen, 1989) in the phylolm package (ver. 2.6); Tung Ho and Ané (2014) in R. For this analysis, we estimated Pagel’s lambda (Pagel, 1997) to evaluate the influence of phylogenetic relationships on the data and tested whether the regression coefficients differed from zero. After the PGLS analyses, we controlled the false discovery rate and estimated the Q-value using the method explained above.

#### The evolutionary history of the *PARP* gene family

Because our analyses revealed the potential role of *PARP* (Poly(ADP-ribose) polymerase) genes in longevity in tree species, we investigated the evolutionary history of the *PARP* gene family in plant species. First, to assess and compare the domain structures of *PARP* genes, we constructed a phylogenetic tree of 189 *PARP* genes from 53 dicot species (Table S7) using the tree explore tool in Dicots PLAZA 4.0 (note that this function in PLAZA is available only for dicot species). Based on the method provided by PLAZA, genes with low sequence similarity were removed from the phylogenetic trees as partial or outlier genes.

Second, we constructed a phylogenetic tree of *PARP* genes from diverse plant species, including angiosperms, gymnosperms, lycophytes, and bryophytes. There were 332 *PARP* genes in the original data set (Table S8). Of 332 genes, 131 were selected by increasing gap-free sites using MaxAlign with a heuristic algorithm (Gouveia-Oliveira et al., 2007) and aligned using the MAFFT online service (Katoh et al., 2018). Then, the phylogenetic tree was constructed using the neighbor-joining method with the Jones–Taylor–Thornton (JTT) substitution model (Jones et al., 1992) and bootstrapping over 1000 trees.

We categorized each *PARP* gene into three groups: *PARP1*, *PARP2*, and *PARP3*, based on different methods in the Dicot and Gymno PLAZA databases. *PARP* genes included in the Dicots PLAZA 4.0 database were categorized following the annotation given in the PLAZA database. We removed the genes categorized as unknown, or genes without detailed annotation, in Dicots PLAZA 4.0. The *PARP* genes included in the Gymno PLAZA 1.0 database were categorized into three different paralogs based on the clustering information in the gymnosperm phylogenetic tree because most of the *PARP* genes included in the Gymno PLAZA 1.0 database showed no annotation.

We constructed the phylogenetic tree for gymnosperms using the same method explained above by extracting 24 gymnosperm *PARP* genes (Table S9 and Figure S5). 88 *PARP* genes in gymnosperms were removed for the phylogenetic tree construction using MaxAlign due to the existence of long gaps in their sequences. These genes were annotated using the Basic Local Alignment Search Tool (BLAST+) (Camacho et al., 2009) against the database, which included 24 sequences of gymnosperm species *PARP* genes used to construct the phylogenetic tree of *PARP* in gymnosperm species. After the annotations, each gene was categorized according to the “best hit” in BLAST. For each paralog of *PARP* gene family, we conducted PGLS regressions and compared the copy number ratios among life forms using the method explained above.

#### The relationship between copy number ratio of *PARP* and growth rate

Our analyses revealed that *PARP* gene family and especially *PARP1* and *PARP2* genes showed significant higher copy number ratios in tree species that generally live longer than herb species. To assess the possibility that the increased copy number of *PARP* is associated with longevity, it is useful to investigate the relationship between copy number ratio of *PARP* and plant lifespan. Because reliable estimation of plant lifespan is very difficult and published maximum tree lifespans are not always supported by scientific evidence (Piovesan and Biondi, 2020), we used growth rate that is inversely related to lifespan of many plant species (Johnson and Abrams, 2009; Black et al., 2008). It has been discussed that long-lived, late successional species typically grow more slowly, invest more resources for defensive compounds and structural support, and maintain lower rates of photosynthesis and respiration than shorter-lived, early successional species (Loehle, 1988).

We successfully collected the data regarding the individual ages and heights in 11 tree species including angiosperms and gymnosperms from the literature (Köstler, 1956; Burns and Honkala, 1990a; 1990b; Liebhard et al., 2003; Bravo-Oviedo et al., 2004) (Table S4). Then, we calculated the average growth rate (the rate of height increment per year) for each species. We collected the data sampled in similar regions (e.g., North America and Switzerland) to align the environmental conditions for tree growth. Because inverse relationship between growth rate and longevity has been argued mainly in tree species, and height growth rate is difficult to obtain in herbs, we applied this analysis only for tree species.

Next, we constructed the phylogenetic tree of 11 tree species for the analysis considering the phylogenetic relationships. We constructed the phylogenetic tree based on amino acid sequences of rbcL and matK using the neighbor-joining method with the JTT substitution model and bootstrapping over 1000 trees by MEGA X (Figure S6).

Finally, to investigate the relationship between the copy number ratio of each type of *PARP* and the growth rate in 11 tree species, we performed PGLS regression using the method explained above. After the regression analyses, we controlled the false discovery rate and estimated the Q-value using the method explained above.

To perform all statistical analyses, we used R ver. 3.6.3 (the R project, http://www.r-project.org/).

## Supplemental information

Document S1. Figures S1–S6

Table S1. The list of DNA repair genes used for the analyses, related to figures 1, 2 and STAR Methods The accession number in Arabidopsis thaliana and ID of gene family in the Dicots PLAZA 4.0 and Gymno PLAZA 1.0 were shown. Each gene was categorized into 11 functional groups.

Table S2. The results of Fisher’s exact test to test enrichment or dilution of each life form in each of significantly different cluster, related to figure 1

Table S3. The results of PGLS regressions in 121 gene families, related to figure 2

Table S4. The list of 11 tree species for analysis of the relationship between the copy number ratio of PARP and the growth rate, related to figure 5, table3 and STAR Methods

Table S5. The species list for the analyses, related to STAR Methods 23 tree species, four shrub species, 15 perennial herb species, 21 annual herb species, and 2 algae species were included. Four shrub species were eliminated from the analyses.

Table S6. The list of species used for the analyses considering the phylogenetic relationships, related to STAR Methods 23 tree species, 15 perennial herb species, and 21 annual herb species were used. Two algae species (*Chlamydomonas reinhardtii* and *Micromonas commoda*) were removed from the analyses because the no sequence data of two algae species was available.

Table S7. The list of 189 PARP genes used for the construction of the phylogenetic tree to compare the domain structures of PARP genes, related to figure 3

Table S8A. The list of 332 PARP genes, related to STAR Methods (A) 131 *PARP* genes used for the construction of the phylogenetic tree.

Table S8B. The list of 332 PARP genes, related to STAR Methods (B) 201 *PARP* genes removed from the construction of the phylogenetic tree by increasing gap-free site using MaxAlign.

Table S9A. The list of PARP genes within gymnosperm species, related to STAR Methods (A) 24 *PARP* genes used for the construction of the phylogenetic tree.

Table S9B. The list of PARP genes within gymnosperm species, related to STAR Methods (B) 88 *PARP* genes removed from the construction of the phylogenetic tree by increasing gap-free site using MaxAlign.
