# Supplementary material for: Copy number analyses of DNA repair genes reveal the role of poly(ADP-ribose) polymerase (PARP) in tree longevity
Source: iScience. 2021 Jun 24;24(7):102779. doi: 10.1016/j.isci.2021.102779 (PMC8271160; doi:10.1016/j.isci.2021.102779)
Supplement: Document S1. Figures S1–S6 [file mmc1.pdf]

iScience, Volume *24*

Supplemental information

Copy number analyses of DNA repair genes reveal the role of poly(ADP-ribose) polymerase (PARP) in tree longevity

Yuta Aoyagi Blue, Junko Kusumi, and Akiko Satake

SUPPLEMENTAL INFORMATION

Supplementary figure legends

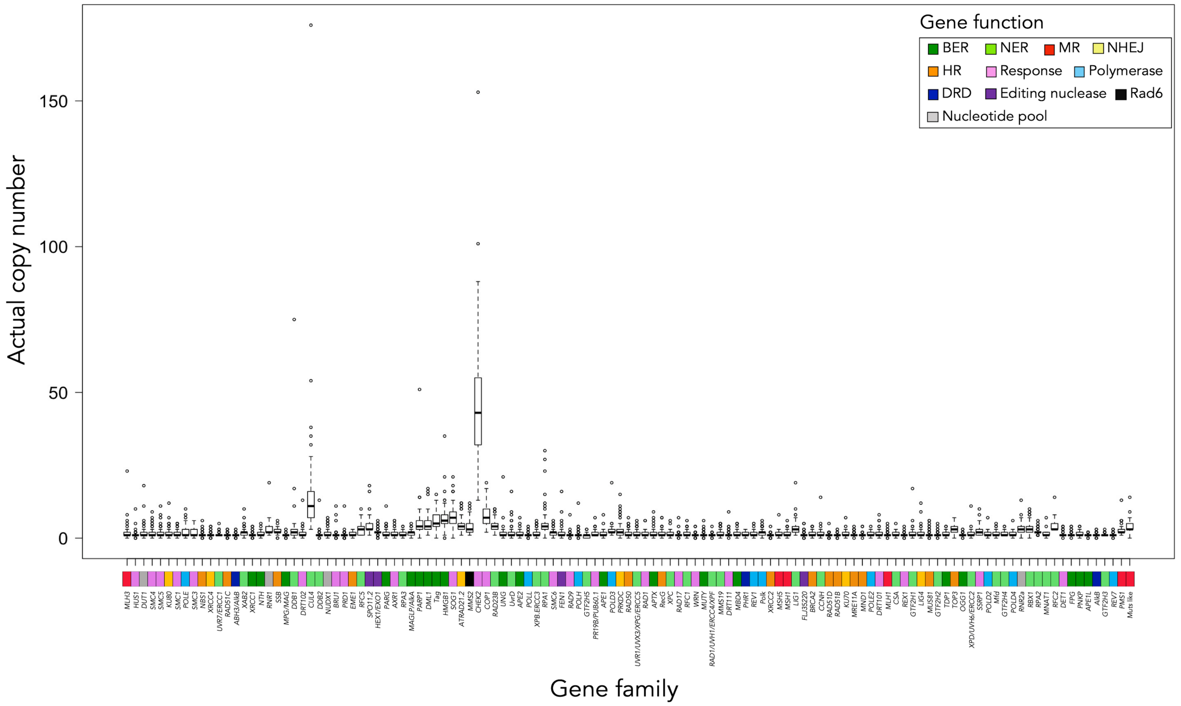

Figure S1. The actual copy number of 121 gene families associated with DNA repair, related to Figure 1. The symbols of the genes within each gene family are shown on the horizontal axis. The horizontal line inside the box showed the median and the length of box showed the interquartile range (range between the 25th to 75th percentiles). The whiskers indicated points within 1.5 times the interquartile rage. The points beyond the whisker range indicated the outliers. The gene families were ordered according to the result of hierarchical clustering. The order of gene families corresponded to the order of gene families in main figure 1a. Each gene family was categorized into one of 11 groups: BER, base excision repair; NER, nucleotide excision repair; MR, mismatch repair; NHEJ, nonhomologous end-joining repair; HR, homologous recombination repair; Response, DNA damage response; Polymerase, DNA polymerase; DRD, direct reversal of damage; Editing nuclease, editing and processing nuclease; Rad6, Rad6 pathway; Nucleotide pool, modulation of nucleotide pool.

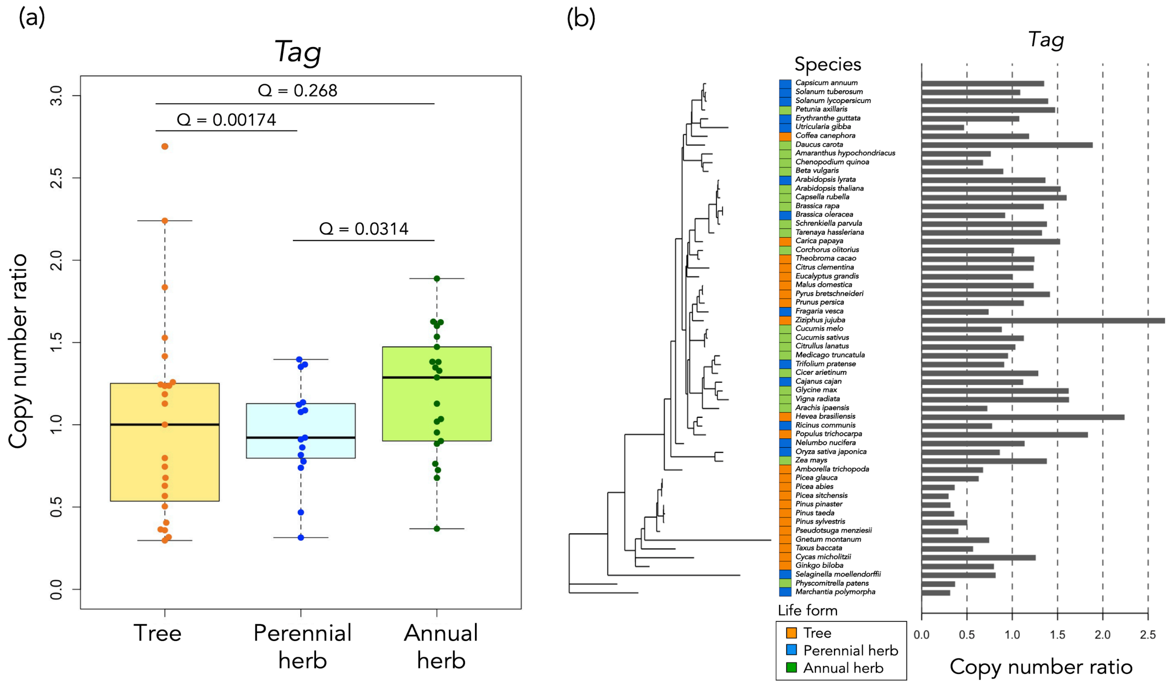

Figure S2. Comparison analysis of the copy number ratio of *Tag* gene families among life forms, related to Figure 2. (a) Box plot of the copy number ratios in different life forms. Tree species had significantly higher copy number ratios than perennial herb species (coefficient = −0.646, standard error = 0.136, *t*-value = −4.75, P-value = 1.46×10^{-5}, Q-value = 0.00174). There was no significant difference between tree species and annual herb species (coefficient = −0.326, standard error = 0.135, *t*-value = −2.41, P-value = 0.0194, Q-value = 0.268). The horizontal line inside the box showed the median and the length of box showed the interquartile range (range between the 25th to 75th percentiles). The whiskers indicated points within 1.5 times the interquartile rage. The points beyond the whisker range indicated the outliers. (b) The phylogenetic relationships of the copy number ratios of the *Tag* gene family. The estimated Pagel's lambda was 0.982.

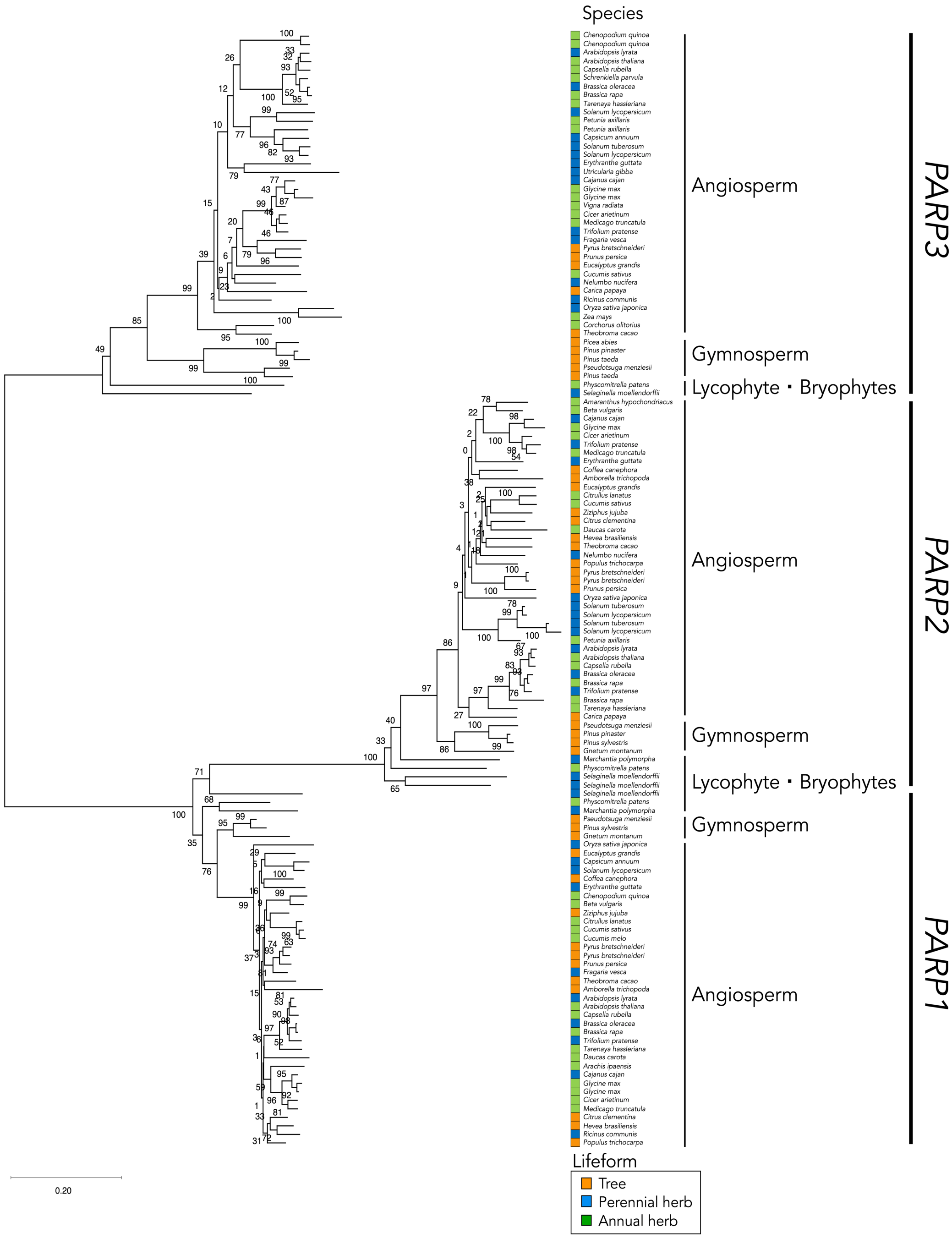

Figure S3. The phylogenetic tree of *PARP* gene family of species in the dataset, related to Figure 3 and Table 2. 131 genes in the species including angiosperms, gymnosperms, lycophyte, and bryophytes. The numbers given on each branch were bootstrap values.

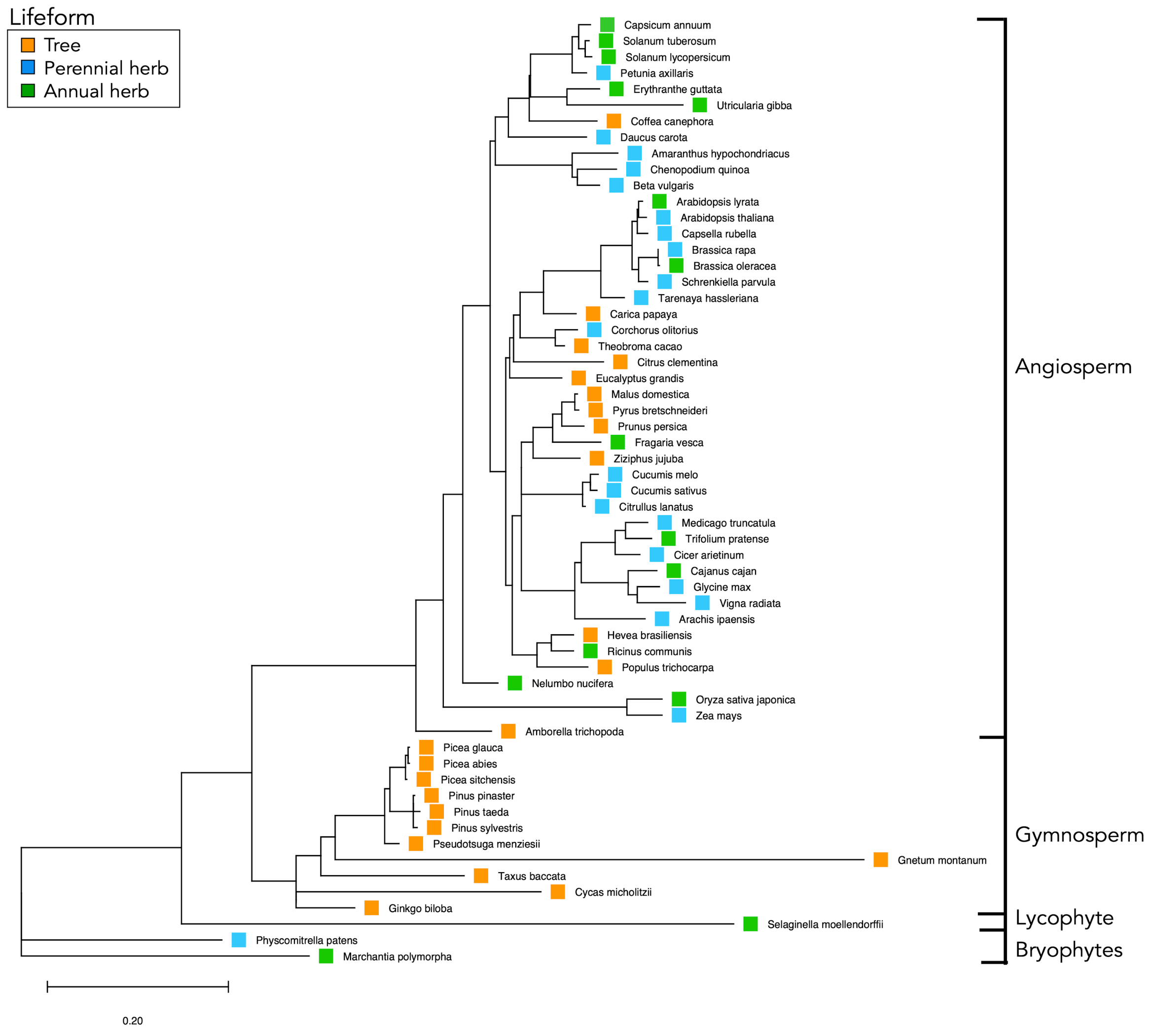

Figure S4. The phylogenetic tree of species for analyses, related to STAR Methods. 23 tree species (orange), 15 perennial herb species (blue), and 21 annual herb species (green) were included.

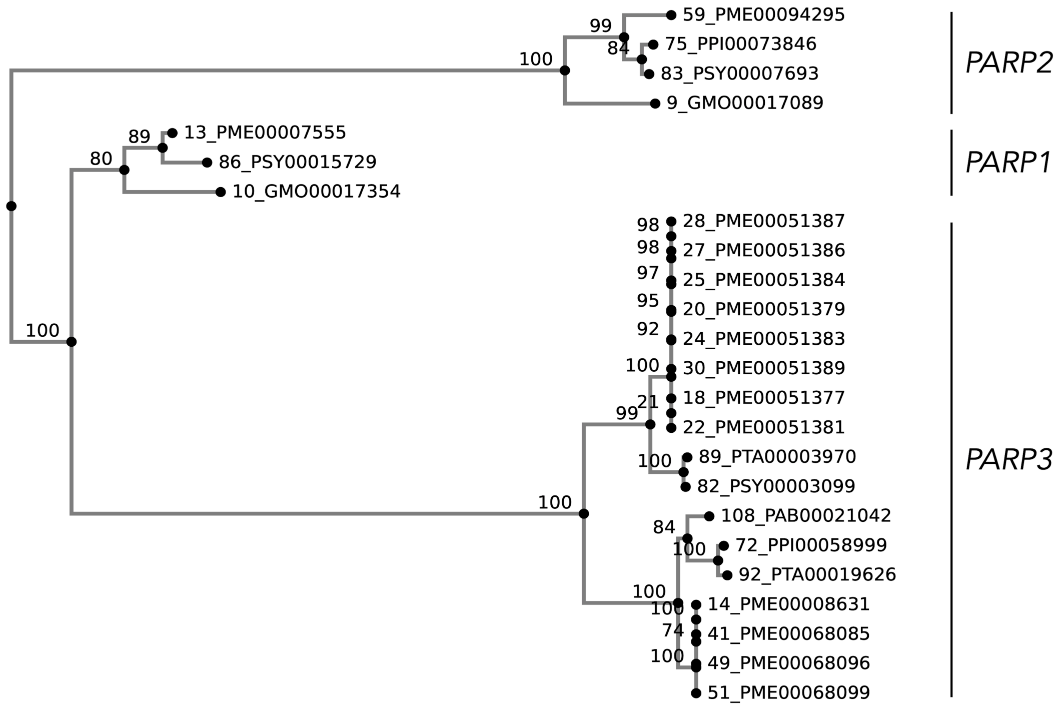

Figure S5. The phylogenetic tree of 24 *PARP* genes within gymnosperm species, related to STAR Methods. 24 *PARP* genes within gymnosperm species were divided into three distinct clades (*PARP1*, *PARP2*, and *PARP3*). The numbers given on each branch were bootstrap values.

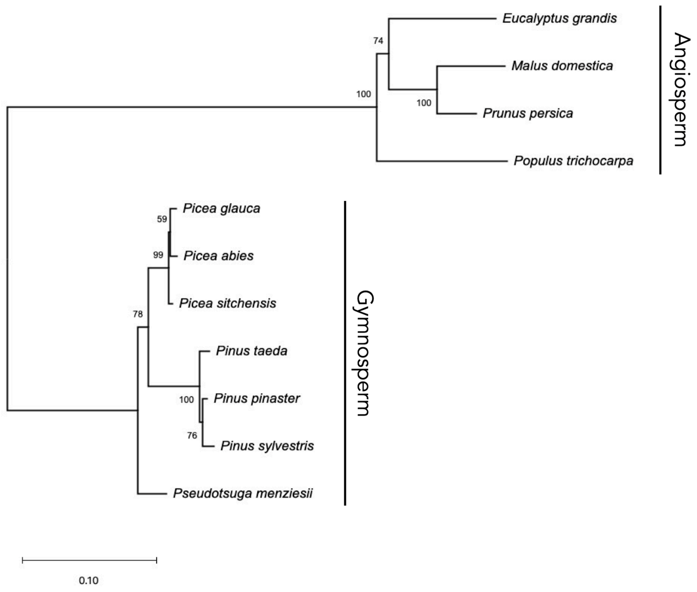

Figure S6. The phylogenetic tree of 11 tree species for analyses of the relationship between the growth rate and the copy number ratio of *PARP*, related to STAR Methods, Figure 5 and Table 3. Four angiosperm and seven gymnosperm species were included. The numbers given on each branch were bootstrap values.
